# Anti-Inflammatory Effects of Neochlorogenic Acid Extract from Mulberry Leaf (*Morus alba L.*) Against LPS-Stimulated Inflammatory Response through Mediating the AMPK/Nrf2 Signaling Pathway in A549 Cells

**DOI:** 10.3390/molecules25061385

**Published:** 2020-03-18

**Authors:** Xiao-han Gao, Sun-dong Zhang, Li-tao Wang, Liang Yu, Xue-lian Zhao, Hai-yan Ni, Yan-qiu Wang, Jian-dong Wang, Chun-hua Shan, Yu-jie Fu

**Affiliations:** 1 College of Life Science, Northeast Forestry University, Harbin 518055, China; 18845127901@163.com; 2 College of Chemistry, Chemical Engineering and Resource Utilization, Northeast Forestry University, Harbin 518055, China; ZSD1426840447@163.com (S.-d.Z.); yl860769292@163.com (L.Y.); 15754634235@163.com (X.-l.Z.); 15556360380@sina.cn (H.-y.N.); 3 College of Forestry, Beijing Forestry University, Beijing 100083, China; Ltwang2018@163.com (L.-t.W.); wyqapple423@163.com (Y.-q.W.); wjdhnnd@163.com (J.-d.W.)

**Keywords:** neochlorogenic acid, anti-inflammatory effect, LPS, inflammatory response, mulberry leaf (*Morus alba L.*), AMPK/Nrf2 signaling pathway

## Abstract

Neochlorogenic acid (nCGA) is a phenolic compound isolated from mulberry leaf (*Morus alba L.*), which possesses multiple pharmacological activities containing antioxidant and anti-inflammatory effects. However, the role of nCGA in the treatment of acute pneumonia and the underlying molecular mechanism are still unclear. Hence, the aim of study is to investigate the anti-inflammatory properties of nCGA on LPS-stimulated inflammation in A549 cells. In the present study, results reported that nCGA without cytotoxicity significantly reduced the production of TNF-α, IL-6, and NO, and further suppressed the proteins of iNOS, COX2, TNF-α, IL-6 expression. Furthermore, nCGA also inhibited NF-κB activation and blocked MAPKs signaling pathway phosphorylation. In addition, we found nCGA significantly increased the expression of HO-1 via activating the AMPK/Nrf2 signaling pathway to attenuate the inflammatory response, whereas this protective effect of nCGA was reversed by pre-treatment with compound C (C.C, an AMPK inhibitor). Therefore, all these results indicated that nCGA might act as a natural anti-inflammatory agent for the treatment of acute pneumonia.

## 1. Introduction

Pneumonia, a current major respiratory disease, is a mortality and morbidity characterized as infectious in clinical cases [1,2]. Acute pneumonia is a common disease in children which is one of the major causes of death in infancy [3,4]. Bacteria and viral infections are the main induction factors that cause lung injury [5]. A recent study reported that the incidence of pneumonia in infants tends to be younger, especially in the first 6 months after birth [6]. Therefore, effective drugs or treatment strategies against acute pneumonia are urgently needed. Normally, lipopolysaccharide (LPS) is a membrane component of Gram-negative bacteria, causing a strong inflammatory response in vivo or in vitro [7,8]. Although the underlying mechanisms have not yet been fully elucidated, accumulating evidence has indicated that LPS could induce pulmonary inflammation and oxidative stress, which contribute to the development of acute pneumonia [9,10]. 

Nuclear factor-kappa B (NF-κB) is a crucial transcriptional factor involved in the regulation of immune and inflammatory response [11]. Generally, upon LPS stimulation, the NF-κB signaling pathway is activated, causing the production of inflammatory factors such as tumor necrosis factor α (TNF-α) and interleukin-6 (IL-6) [12]. LPS induced NF-κB p65, dissociating from IκB and translocating into the nucleus, which bound to the promoter of target genes and activated transcription and further induced the expression of inflammatory cytokines [13,14]. Meanwhile, oxidative stress also played a key role in the development of lung pathogenesis, which aggravated cellular and lung tissues injury in cells and animals [15,16]. Additionally, studies indicated that mitogen-activated protein kinase (MAPK) was activated, and in turn, activated a series of genes expression such as apoptosis and inflammatory genes [17]. Thus, inhibition of these signaling pathways may attenuate inflammatory responses and oxidative damage, which also might serve as potential therapeutic targets in acute pneumonia.

It is well-known that nuclear factor erythroid 2-related factor (Nrf2) is the main redox sensing transcription factor, which plays an important role in the regulation of oxidative stress [18,19]. Normally, under oxidative stress stimulation, Nrf2 is activated, released from the Keap1-Nrf2 complex, and transferred from cytoplasm to nucleus. It further initiated transcription of various anti-oxidative genes including Heme oxygenase-1 (HO-1) [20]. However, more evidence indicated that HO-1 induction blocked LPS-induced inflammation responses by activation of Nrf2 signaling in vitro and in vivo [21]. Then, AMP-activated protein kinase (AMPK) was also a widely known energy sensor involved in LPS-induced inflammatory responses and oxidative stress, and these processes were improved under AMPK activation conditions [22,23,24]. Moreover, recent studies reported that AMPK activation could facilitate the nuclear translocation of Nrf2 and upregulate HO-1 expression to attenuate LPS-induced cellular injury [23,25]. Hence, activation of AMPK and Nrf2 are benefit to anti-inflammatory responses among acute pneumonia occurance.

Mulberry leaf (*Morus alba L.*) is one of the Chinese traditional edibile plants, which is extensively consumed in various forms such as tea, biscuits, beverage in our daily life [26,27]. Mulberry leaf is rich in active compounds including alkaloids, flavonoids, polysaccharides, and phenolic compounds, which possesses multiple pharmacological activities containing anti-thrombotic, anti-diabetic, anti-hypertensive, anti-bacterial, anti-inflammatory, and anti-aging [28,29]. Indeed, recent studies demonstrated that its leaves are used to treat type 2 diabetes, cardiovascular disorders, urinary system, anti-tumor, and so on [30]. Neochlorogenic acid (nCGA), as an isomer of chlorogenic acid (CGA), was a phenolic compound isolated from mulberry leaf (*Morus alba L.*), which also had functions on scavenging free radicals, antioxidant, and anti-inflammatory to alleviate oxidative stress consistent with CGA [31,32]. Its anti-inflammatory effects have been well-identified, and it have been helpful in the treatment of rheumatoid arthritis and inflammatory bowel disease [32,33]. However, evidence of the role of nCGA in the treatment of acute pneumonia is lacking and the underlying molecular mechanisms are still unclear. Hence, the present study investigated the anti-inflammatory effect of nCGA on A549 cells to provide basic research for nCGA applications in pneumonia therapeutics.

## 2. Results

### 2.1. Effects of nCGA and LPS on Cell Viability in A549 Cells

**Figure 1:**
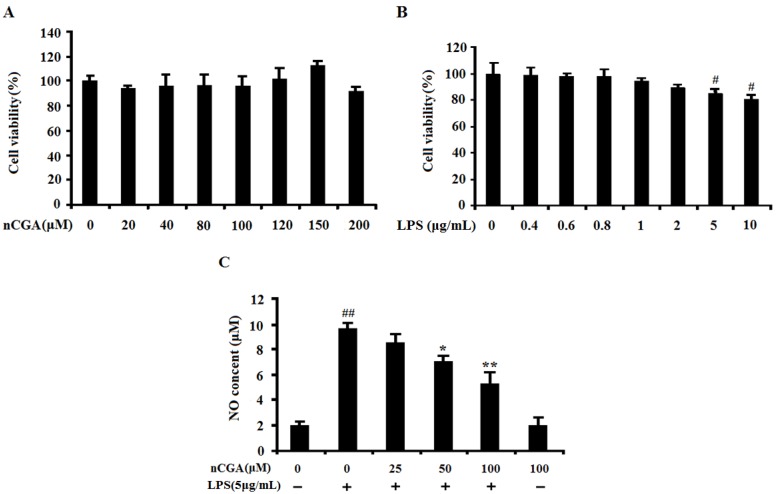
Effects of Neochlorogenic acid (nCGA) on cytotoxicity of A549 cells. (**A**) A549 cells were incubated with various concentrations of nCGA (0 to 200 μM) for 24 h, the cytotoxicity was measured by MTT assay. (**B**)A549 cells were treated with different concentration of LPS for 24 h, the cell viability was measured by MTT assay, the optimum concentration of LPS was selected for further study. (**C**) A549 cells were pretreated with or without various concentrations of nCGA (0, 25, 50, 100 μM) for 2 h before LPS (5 μg/mL) stimulation for another 24 h, the level of NO was measured in the culture medium by Griess reagents. The values represent the mean ± SD of three independent experiments, significant differences between different groups. #*p* < 0.05, ##*p* < 0.01 vs. control group, * *p* < 0.05, ** *p* < 0.01 vs. LPS alone.

The effects of nCGA and LPS on cell viability were investigated on A549 cells using 3-[4,5-dimehyl-2-thiazolyl]-2,5-diphenyl-2H-tetrazolium bromide (MTT) method. Cells were pretreated with different concentrations of nCGA and LPS for 24 h, and the percentage of cell viability in A549 cells were shown in Figure 1. The result demonstrated that nCGA had no significant effect on A549 cells viability with non-cytotoxic (Figure 1A). In addition, the result also showed that LPS slightly affected cell growth in a dose-dependent manner with low toxicity. To avoid the cytotoxic, the usage of LPS at a concentration of 5 μg/mL was chosen for the best experimental condition (Figure 1B). In addition, nitric oxide (NO), a kind of inflammatory mediator, is expressed only after exposure to pro-inflammatory conditions. As shown in Figure 1C, the results suggested that LPS significantly induced NO production compared with the control group, whereas this result was improved by nCGA treatment. Therefore, these results excluded the possibility that inflammatory responses were caused by the cytotoxicity of nCGA in A549 cells.

### 2.2. Effects of nCGA on Inflammatory Cytokines Production in LPS-Stimulated A549 Cells

**Figure 2:**
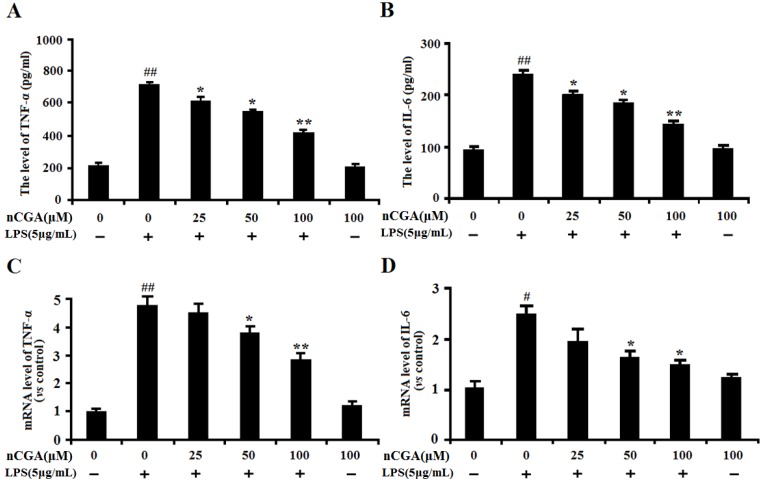
Effects of nCGA on inflammatory cytokines production in Lipopolysaccharide (LPS)-stimulated A549 cells. A549 cells were pretreated with or without various concentrations of nCGA (0, 25, 50, 100 μM) for 2 h before LPS (5 μg/mL) stimulation for another 24 h. (**A**,**B**) The levels of TNF-α, IL-6 were detected in the culture medium by ELISA kits. (**C**,**D**) The mRNA levels of TNF-α and IL-6 were detected by RT-PCR. The values represent the mean ± SD of three independent experiments, significant differences between different groups. # *p* < 0.05, ## *p* < 0.01 vs. control group, * *p* < 0.05, ** *p* < 0.01 vs. LPS alone.

In this study, to determine the optimal time for LPS-induced inflammatory response, the levels of TNF-α, IL-6 and NO were measured after LPS stimulated A549 cells for 3, 6, 12, 18, 24 h. As showed in Figure S1, the results showed that LPS significantly activated inflammatory response by increasing the inflammatory cytokines. To detect the anti-inflammatory effect of nCGA under LPS-stimulated in A549 cells, cells were pretreated with or without different concentrations of nCGA for 2 h before LPS stimulation for another 24 h. As shown in Figure 2A,B, the results showed that pre-treatment with nCGA dose-dependently decreased the production of TNF-α and IL-6, compared with LPS treatment alone. Additionally, the RT-PCR assay found that nCGA also obviously decreased the mRNA levels of *TNF-α* and *IL-6* in a dose-dependent manner. All data demonstrated that nCGA markedly attenuated LPS-stimulated inflammatory responses in A549 cells.

### 2.3. Effects of nCGA on the Expression of Inflammatory-Related Proteins in LPS-Stimulated A549 Cells

**Figure 3:**
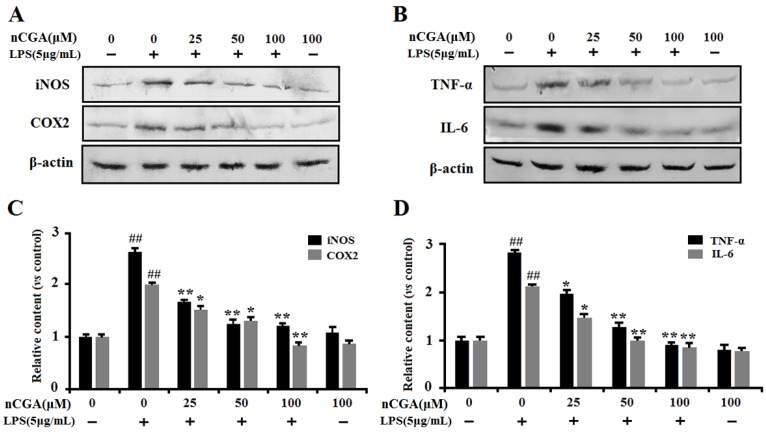
Effects of nCGA on the expression of inflammatory-related proteins in LPS-stimulated A549 cells. Cells were pretreated with or without various concentrations of nCGA (0, 25, 50, 100 μM) for 2 h before LPS (5 μg/mL) stimulation for another 24 h. (**A**,**B**) To further demonstrate its anti-inflammatory effects, the proteins of iNOS, COX2, TNF-α, and IL-6 expression were determined by Western blot analysis. (**C**,**D**) The quantitative analysis was performed by normalization with β-actin. The values represent the mean ± SD of three independent experiments, significant differences between different groups. #*p* < 0.05, ## *p* < 0.01 vs. control group, * *p* < 0.05, ** *p* < 0.01 vs. LPS alone.

To further confirm its anti-inflammatory effect, the inflammatory-related proteins of iNOS, COX2, TNF-α, and IL-6 expression were preformed using Western blotting analysis. As shown in Figure 3A,B, the results showed that LPS significantly induced inflammatory-related proteins expression of TNF-α, IL-6, iNOS and COX2, whereas pretreatment with nCGA could obviously inhibit such effect in A549 cells. The quantitative analysis of protein expression was shown in Figure 3C,D. Hence, all results indicated that nCGA alleviated LPS-stimulated inflammatory responses in A549 cells by inhibiting the proteins of iNOS, COX2, TNF-α, and IL-6 expression.

### 2.4. nCGA Attenuated LPS-Stimulated Inflammatory Responses via Inhibiting MAPK Phosphorylation and NF-κB Activation in A549 Cells

**Figure 4:**
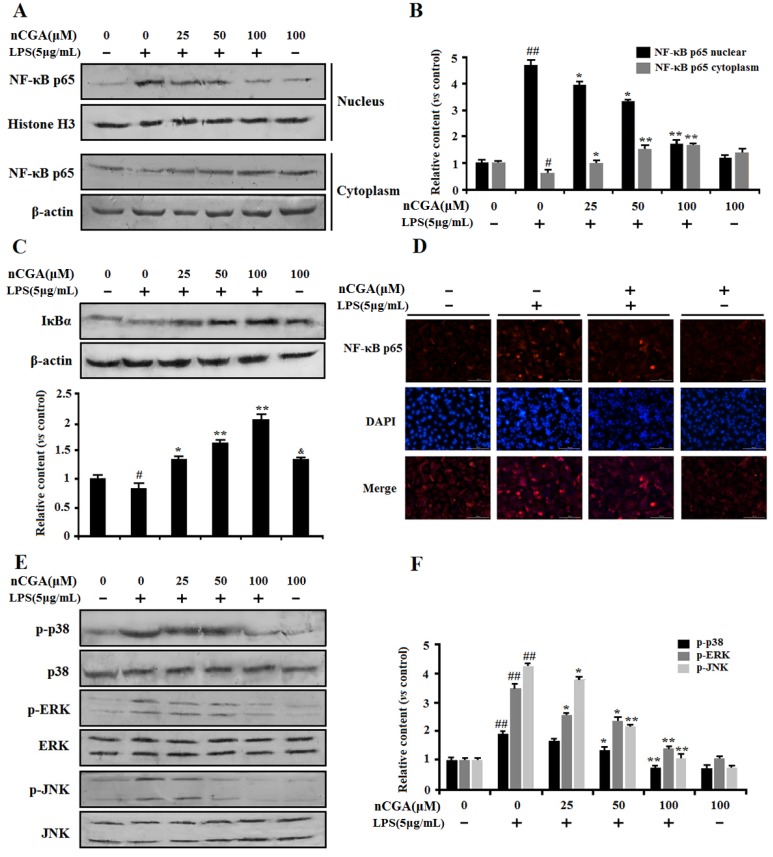
Effects of nCGA on the NF-κB and MAPK signaling pathway in A549 cells. Cells were pretreated with or without various concentrations of nGCA (0, 25, 50, 100 μM) for 2 h before LPS (5 μg/mL) stimulation for another 6 h. (**A**,**B**) nCGA inhibited the NF-κB activation, the level of NF-κB p65 in the nucleus and cytoplasm were detected using Western blot analysis. (**C**) Western blot analysis showed the protein expression levels of IκB. (**D**) To verify the above results, the expression of the NF-κB p65 subunit in the nucleus was measured using immunofluorescence assay (NF-κB p65 subunit and nuclei were viewed red and blue, respectively). (**E**,**F**) The expression of total p38, ERK, JNK, and phosphorylation were detected by Western blot analysis. The values represent the mean ± SD of three independent experiments, significant differences between different groups. # p < 0.05, ## p < 0.01 vs. control group, * *p* < 0.05, ** *p* < 0.01 vs. LPS alone, ^&^ *p* < 0.05, nCGA treated group vs. control group.

Normally, the NF-κB signaling pathway is important in mediating inflammatory responses, which is located in the cytoplasm with inhibitory protein IκB. Upon stimulation with LPS, NF-κB p65 was dissociated from IκB and translocated into the nucleus. To investigate the anti-inflammatory effect of nCGA on the NF-κB pathway, the proteins of NF-κB p65 expression in nucleus and cytoplasm were detected (Figure 4A,B). The result showed that nCGA significantly inhibited NF-κB p65 nuclear translocation. At the same time, as shown in Figure 4C, the protein of IκB degradation was inhibited to further demonstrate that nCGA could effectively suppress NF-κB activation. To further demonstrate this process, we measured the expression of the NF-κB p65 subunit in the nucleus using immunofluorescence assay. As shown in Figure 4D, the image showed that LPS markedly activated NF-κB p65 nuclear translocation, this phenomenon was improved after pretreatment with nCGA. On the other hand, a recent study reported that MPAK mediated intracellular inflammatory signal transduction and oxidative stress in acute lung injury after LPS induction. Effect of nCGA on MAPK signaling regulation was detected through measuring the expression of p38 MAPK, ERK1/2, and JNK. As shown in Figure 4E,F, the Western blot analysis performed that the phosphorylation of p38 MAPK and ERK1/2 were increased in LPS-induced alone group, whereas nCGA significantly inhibited p38 MAPK and ERK1/2 phosphorylation in a dose-dependent manner. Thus, these results showed that the anti-inflammatory action of nCGA was related to blocking nuclear transfer of NF-KB and inhibiting phosphorylation of p38 MAPK and ERK1/2.

### 2.5. Effects of nCGA on Regulation of Nrf2 and AMPK Against LPS-Induced Inflammatory Responses in A549 Cells

**Figure 5:**
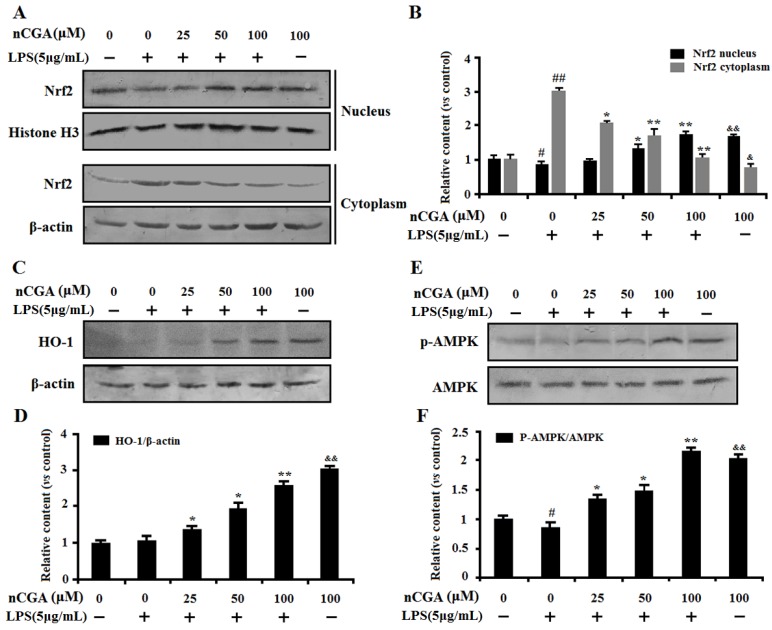
Effects of nCGA on regulation of Nrf2 and AMPK against LPS-induced inflammatory responses in A549 cells. Cells were pretreated with or without various concentrations of nCGA (0, 25, 50, 100 μM) for 2 h before incubation with LPS (5 μg/mL) for another 24 h. (**A**,**B**) The nuclear and cytoplasmic Nrf2 were measured by Western blotting and the quantitative analysis were performed by normalization with Histone H3 and β-actin, respectively. (**C**,**D**) Western blot analysis showed the protein expression level of HO-1 was normalized with β-actin. (**E**,**F**) To investigate the effect of nCGA on AMPK signaling pathway, the level of p-AMPK was determined by Western blot analysis. The values represent the mean ± SD of three independent experiments, significant differences between different groups. # *p* < 0.05, ## *p* < 0.01 vs. control group, * *p* < 0.05, ** *p* < 0.01 vs. LPS alone, ^&^ *p* < 0.05, ^&&^ *p* < 0.01 nCGA treated group vs. control group.

It is well-known that Nrf2, the main redox sensing transcription factor, plays an important role in the regulation of oxidative response and inflammatory responses. Recent studies indicated that HO-1 induction could block LPS-induced inflammation by activation of Nrf2 signaling. To further determine the anti-inflammatory mechanism of nCGA in A549 cells, we assessed the effect of nCGA on Nrf2 expression in the nucleus and cytoplasm by Western blot analysis. As shown in Figure 5A,B, results reported that nCGA significantly promoted Nrf2 nuclear translocation compared with treatment with LPS alone. In addition, the similar result of HO-1 expression was increased in a dose-dependent manner after nCGA treatment (Figure 5C,D). Meanwhile, more studies showed that AMPK involved in LPS-induced inflammatory response and this process was improved upon AMPK activation. To investigate the effect of nCGA on the AMPK signaling pathway, the level of AMPK was determined by Western blot analysis. As shown in Figure 5E,F, the result indicated that nCGA dose-dependently activated the phosphorylation of AMPK in A549 cells exposure with or without LPS. Taken together, these findings demonstrated that nCGA protected cells against LPS-stimulated inflammatory responses via upregulating the Nrf2 signaling pathway and activating AMPK phosphorylation.

### 2.6. The Importance of AMPK Activation in the Process of nCGA Against LPS-Induced Inflammatory Responses

**Figure 6:**
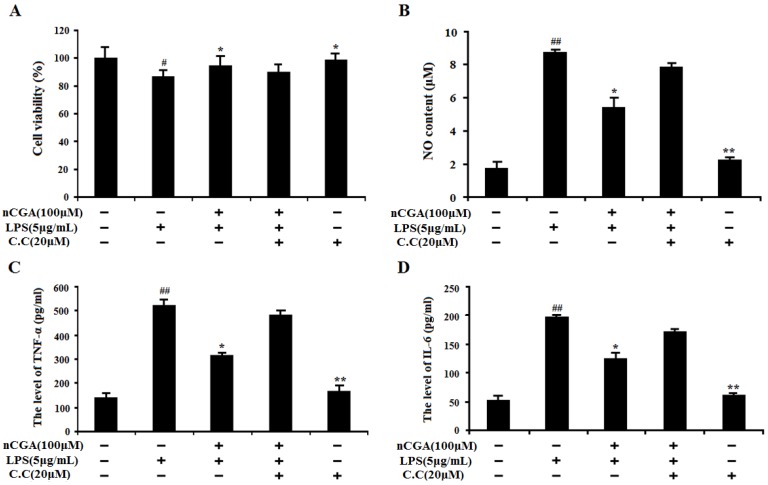
Effect of AMPK activation in the process of nCGA against LPS-induced inflammatory responses. A 549 cells were pre-incubated with presence or absence C.C (20 μM) for 1 h, and then treated with 100 μM nCGA for 2 h before exposure with LPS. (**A**) The cell viability was detected by MTT assay. (**B**) NO content. (**C**,**D**) The levels of TNF-α and IL-6 were measured by ELISA assay. The values represent the mean ± SD of three independent experiments, significant differences between different groups. # *p* < 0.05, ## *p* < 0.01 vs. control group, * *p* < 0.05, ** *p* < 0.01 vs. LPS alone.

To evaluate the importance of AMPK activation, the compound C (C.C, an AMPK inhibitor) was used in this investigation. A549 cells were pre-incubated with presence or absence C.C for 1 h, and then treated with nCGA for 2 h before exposure with LPS. As shown in Figure 6A, the result showed that 20 μM C.C had no cytotoxicity to A549 cells. Interestingly, nCGA significantly decreased the NO content against LPS-induced inflammatory response, whereas this inhibition effect was reversed by C.C (Figure 6B). In addition, as shown in Figure 6C,D, the similarly results suggested that C.C obviously reversed the inhibition effect of nCGA on TNF-α and IL-6 levels in LPS-induced A549 cells. Therefore, all data demonstrated that AMPK activation played a key role in the protective effect of nCGA against LPS-induced inflammatory response in A549 cells.

## 3. Discussion

Acute pneumonia is a prevalent respiratory disease, susceptible to viral and bacterial infections and easily occurs in infancy. The disease is characterized by severe symptoms and complex complications along with high morbidity and mortality [3,5]. Therefore, natural compounds that can improve pulmonary inflammatory response with low-side effects have received extensive attention. Previous studies demonstrated that phenolic compounds had strong antioxidant activity, which could scavenge free radicals and prevent the inflammatory diseases through suppressing oxidative stress [34,35]. In the present study, nCGA is a natural phenolic compound isolated from mulberry leaf, its anti-inflammatory activity was further investigated. The present study firstly demonstrated that nCGA played important roles in preventing and treating acute pneumonia and revealed an underlying mechanism of LPS-induced inflammatory response in A549 cells.

Inflammatory response is a common pathological process, which reflects the fight between host and inflammatory factors. LPS is a major constituent of the outer membrane of Gram-negative bacteria, which is able to induce inflammatory response in all types of cells [8,36]. Moreover, LPS as an inflammatory inducer that could trigger pro-inflammatory cytokines and mediators production, such as TNF-α, IL-6, and nitric oxide (NO), respectively [7,9]. Accumulating studies reported that these cytokines and mediators played important roles in the development and progression of inflammatory response [37]. Thus, inhibition of pro-inflammatory cytokines production has been regarded as a helpful therapeutic strategy. In our study, we found LPS could significantly stimulate the production of TNF-α, IL-6, and NO, which was consistent with previous studies. In addition, the data showed that proteins of iNOS, COX2, TNF-α, and IL-6 expression were upregulated in A549 cells upon LPS stimulation, whereas treatment with nCGA obviously reduced pro-inflammatory production and suppressed the expression of iNOS, COX2, TNF-α, and IL-6 in a dose-dependent manner. All these results supported that nCGA exerted protective effects against LPS-induced inflammatory response in A549 cells.

NF-κB is a well-known transcription factor associated with inflammatory response, oxidative stress, and the apoptosis process [11]. Indeed, more studies reported LPS could trigger the NF-κB signaling pathway activation, which mediated the expression of pro-inflammatory genes such as *TNF-α* and *IL-6* [7,12]. Therefore, inhibition of NF-κB activation is an important strategy in the treatment of inflammatory response. Our study investigated the effect of nCGA on NF-κB activation. The results showed that nCGA significantly reduced IκB degradation and inhibited NF-κB p65 phosphorylation. Furthermore, the results also suggested that treatment with nCGA also obviously suppressed LPS-induced NF-κB nuclear translocation through immunofluorescence and Western blot analysis. On the other hand, MAPKs signaling pathway containing ERK, JNK, and p38 MAPK, which played a central role involved in inflammatory response and cell survival [17,38]. For certain, recent studies indicated the phosphorylation of MAPKs were activated in LPS-stimulated RAW 264.7 cells. Meanwhile, phosphorylation of MAPKs was helpful to promote NF-κB activation and aggravate pro-inflammatory cytokines and mediators secretion [39]. Hence, we verified the effect of nCGA on the MAPKs signaling pathway and the results revealed nCGA markedly inhibited LPS-stimulated ERK, JNK, and p38 MAPK phosphorylation in A549 cells, and these manifestations had no significant changes after treatment with nCGA alone compared with the control group. All these results indicated that the anti-inflammatory effect of nCGA was mostly associated with blocking NF-κB activation and suppressing MAPKs phosphorylation.

To further uncover whether the underlying molecular mechanisms of nCGA to alleviate LPS-induced inflammatory response in A549 cells is related to its antioxidant capacity, the study investigated the effect of nCGA on oxidation-associated genes such as Nrf2, which was a transcription factor involved in maintaining cellular redox homeostasis [40]. Previous studies suggested that Nrf2 played a vital role in the production of inflammatory cytokines and mediators via scavenging free radical against oxidative stress [18,19,41]. Moreover, this regulatory mechanism is achieved through increasing Nrf2 nuclear transfer and binding to ARE sites, which initiates transcription of HO-1 to eliminate ROS and counteract oxidative damage [20,42]. Therefore, our results demonstrated that nCGA significantly increased the nuclear level of Nrf2 and further upregulated HO-1 expression in a dose-dependent manner via Western blot analysis. Furthermore, AMPK is an important gene in the protein kinase cascade which participates in the pathogenesis of various diseases [22,24]. More studies found that AMPK could mediate various downstream molecules expression including Nrf2 and HO-1, which ameliorated acute and chronic pulmonary, inflammatory, and pathophysiological processes [23,25]. In addition, AMPK signal activation could diminish nuclear translocation of NF-κB and further attenuate LPS-stimulated inflammatory response [43,44]. Our study suggested that nCGA obviously activated the AMPK signaling pathway and increased AMPK phosphorylation compared with treatment with LPS alone. To verify the importance of AMPK activation in the anti-inflammatory effect of nCGA, C.C, an AMPK inhibitor could notably reverse the protective effect of nCGA against LPS-stimulated inflammatory response. Therefore, all results likely provide a new strategy for nCGA to prevent or treat acute pneumonia.

In conclusion, the present study demonstrated that nCGA inhibited LPS-induced inflammatory response in A549 cells, it reflected that nCGA could be a natural anti-inflammatory agent for application in acute pneumonia. The results showed that nCGA could reduce pro-inflammatory cytokines production and inhibit NF-κB activation and MAPK phosphorylation. And to further demonstrate that the anti-inflammatory effect of nCGA is associated with activating AMPK and the Nrf2 signaling pathway. Taken together, all data concluded that nCGA, a natural active compound, might be a potential candidate to prevent acute pneumonia. In addition, the limitation of this study is that the anti-inflammatory effect of nCGA on other lung cell lines has not been fully investigated, and the biosafety assessment might be better considered as well.

## 4. Materials and Method

### 4.1. Reagents and Chemicals

**Figure 7:**
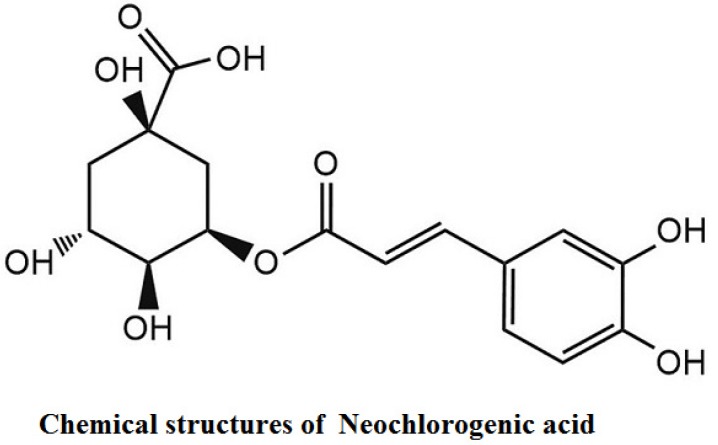
The chemical structure of nCGA.

Neochlorogenic acid (nCGA, purity >98%) was isolated from mulberry leaf (*Morus alba L.*), and the chemical structure was identified in our laboratory [45] (Figure 7). A 10 mM stock solution of nCGA was prepared in dimethyl sulfoxide (DMSO).

Lipopolysaccharide (*Escherichia coli* 055:B5), MTT and dimethylsulfoximine (DMSO) were purchased from Sigma (St. Louis, MO, USA). Fetal bovine serum (FBS) and RPMI 1640 medium were obtained from HyClone (Logan, Utah, U.S.). Human ELISA kits were purchased form Biolegend (San Diego, CA, USA). Compound C (AMPK inhibitor) was purchased from Sigma (St. Louis, MO, USA). Primary antibodies against phospho-ERK1/2, ERK1/2, phospho-p38 MAPK, p38 MAPK, JNK, phospho-JNK, p65/p-p65, IκBα, and β-actin were purchased from Beyotime Institute of Biotechnology (Beijing, China), primary antibodies against TNF-α, IL-6, iNOS, and COX2 were obtained from Biolegend (San Diego, CA, USA). Second antibodies and antibiotics (100 U/mL penicillin and 100 μg/mL streptomycin) were obtained from Beyotime Institute of Biotechnology (Beijing, China). All other chemicals were of reagent grade.

### 4.2. Cell Culture

Human lung adenocarcinoma cell line A549 cells were purchased from American Type Culture Collection (USA), which used as a model of lung tissue cells considering the effect of LPS induced pulmonary inflammation. Cells were cultured in RPMI 1640 medium supplemented with 10% FBS and antibiotics (100 U/mL penicillin and 100 μg/mL streptomycin). Then cells were kept in a humid atmosphere incubator containing 5% CO_2_ and 37 °C.

### 4.3. Cell Viability Assay

For the evaluation of LPS and nCGA on cytotoxicity, cell viability was measured by the MTT assay. A549 cells were seeded in 96-well plates at a density of 1 × 10^4^ cells per well. After incubation overnight with medium, the cells were treated with various concentrations of LPS and nCGA for 24 h, and DMSO was added as control. Then, 20 μL of MTT (5 mg/mL) was added into per well and incubated for 4 h at 37 °C. After the medium was removed completely, 100 μL DMSO were added into cell cultures and shaken for 10 min. The absorbance was measured at a wavelength of 570 nm using an Ultra Microplate Reader (TECAN, Switzerland).

### 4.4. Nitrite Assay

The nitrite assay was carried out to detected the NO production according to the manufacturer’s instructions. Briefly, A549 cells were planted in 24-well plates and incubated for overnight, then cells were treated with different concentrations of nCGA for 2 h before exposure with LPS. After incubation for 24 h, each well culture supernatant was collected into 96-well plates and was mixed with Griess reagent. The absorbance was measured at 540 nm by using an Ultra Microplate Reader (TECAN, Switzerland). The concentration of NO was determined using NO standard. Three replicates were carried out for each sample.

### 4.5. Enzyme-Linked Immunosorbent Assay

A549 cells were planted into 24-well plates at a density of 2 × 10^5^ cells for overnight growth. Then, cells were pretreated with or without various concentrations of nCGA for 2 h before treatment with LPS. After incubation for 24 h, the culture media was collected and determined the levels of cytokines for TNF-α and IL-6 using enzyme-linked immunosorbent assay according to the manufacturer’s protocols. Three replicates were conducted for each sample.

### 4.6. Quantitative Real-Time PCR

Cells were planted in 6-well plates and grown for 24 h before pretreated with or without different concentrations of nCGA for 2 h, After stimulation with LPS for 24 h, the total RNA was extracted using the Trizol Reagent (Invitrogen, Carlsbad, USA) according to the manufacturer’s instruction. The concentration and purity of RNA were measured by NanoDrop™ 2000c Spectrophotometer (Thermo Scientific, Waltham, MA, USA). The cDNA synthesis was carried out following the protocol of the Revertaid First Strand cDNA Synthesis Kit (Thermo Scientific, USA). Then, the relative genes expressions were quantitated using the LightCycler^®^ 96 Real-Time PCR Detection System (Roche, Switzerland). For PCR analysis, the PCR primers were synthesized by Sangon Biotech (Shanghai, China) and the sequences are shown in Table 1. The target genes expression in different groups was measured by using the formula 2^−ΔΔCT^ method, relative to the control group.

### 4.7. Immunofluorescence Assay

Immunofluorescence assay was used to detect whether the NF-κB p65 was transferred into the nucleus using the Nuclear NF-κB Translocation Assay Kit (Beyotime Institute of Biotechnology, Beijing, China). In brief, A549 cells were pretreated with or without nCGA before stimulation with LPS for 6 h, after washing and fixing, then cells were incubated with immunostaining blocking buffer for 1 h to block non-specific binding. After that, cells were incubated with the primary NF-κB p65 antibody for 1 h at room temperature, and further incubated for 1 h after adding Cy3-conjugated secondary antibody. Then, cells were stained with DAPI for 5 min before observation. NF-κB p65 subunit and nuclei were viewed red and blue under the laser confocal microscope, respectively. Merging red and blue images produced purple fluorescence in areas of co-localization.

### 4.8. Western Blot Analysis

A549 cells were seeded in 6-well cell culture plates at a density of 1 × 10 ^6^ cells for overnight growth. Then, cells were pretreated with or without different concentrations of nCGA for 2 h before exposure with LPS. Cells were washed twice with ice-cold PBS and lysed with RIPA buffer containing 1 mM phenylmethanesulfonyl fluoride (PMSF) and 1× protease inhibitor cocktail. The cell lysates were centrifuged at 12,000 rpm for 10 min and the supernatant was collected. The protein concentration was measured by BCA protein assay kit (Sigma, USA), separated on polyacrylamide gel electrophoresis (12%) and transferred on apolyvinylidene difluoride (PVDF) membranes, which were blocked at room temperature for 2 h with blocking solution (1% BSA in PBS plus 0.05% Tween-20) to reduce nonspecific binding. Then, the membranes were incubated with primary antibodies (1:500) at 4 °C overnight, and membranes were incubated with the secondary antibody (1:1000) at room temperature for 2 h. Finally, detection was performed using the BCIP/NBT Alkaline Phosphatase Color Development Kit (Beyotime Institute of Biotechnology, Beijing, China) according to the manufacturer’s instructions. Bands were then recorded by a digital camera (Canon, EOS 350D, Tokyo, Japan).

### 4.9. Preparation of Cytoplasmic and Nuclear Protein Extracts

The extraction of nuclear and cytoplasmic proteins were carried out by using the Nuclear and Cytoplasmic Extraction Kit from Beyotime Institute of Biotechnology (Beijing, China). Extraction processes were performed strictly according to the manufacturer’s instructions. Then, cytoplasmic and nuclear proteins were measured by Western blot analysis, as mentioned above.

### 4.10. Statistical Analysis

All data are expressed as mean values ± standard deviation. Differences between three independent experiments were calculated by one-way ANOVA. Statistical analysis was performed using one-way ANOVA, followed by post-hoc tests (using least significant difference test, LSD-t) for multi-group comparisons. *P* < 0.05 was considered as statistically significant.

## Figures and Tables

**Table 1:** The sequences of the primers.

	Forward Primer	Reverse Primer
*TNF-α*	5′-CCAACGGCATGGATCTCAA-3′	5′-TTGACGGCAGAGAGGAGGT-3′
*IL-6*	5′-TCCATCCAGTTGCCTTCTTG-3′	5′-AAGCCTCCGACTTGTGAAGT-3′
*β-actin*	5′-CTCCATCCTGGCCTCGCTGT-3′	5′-GCTGTCACCTTCACCGTTCC-3′

## Supplementary Materials

The following are available online, Figure S1: Effects of LPS on inflammatory responses in time-dependence in A549 cells.

## Abbreviations

MTT	3-[4–dimethylthiazol-2-yl]-2,5-diphenyltetrazolium bromide
LPS	lipopolysaccharide
nCGA	neochlorogenic acid
AMPK	AMP-activated protein kinase
MAPK	mitogen-activated protein kinase
Nrf2	nuclear factor-crythroid 2-related factor
HO-1	heme oxygenase-1
TNF-α	tumor necrosis factor α
IL-6	interleukin-6
iNOS	nitric oxide synthase
COX2	Cyclooxygenase 2
NO	nitric oxide
C.C	Compound C
