# Supplementary material for: Anti-Inflammatory Effects of Neochlorogenic Acid Extract from Mulberry Leaf (Morus alba L.) Against LPS-Stimulated Inflammatory Response through Mediating the AMPK/Nrf2 Signaling Pathway in A549 Cells
Source: Molecules. 2020 Mar 18;25(6):1385. doi: 10.3390/molecules25061385 (PMC7144357; doi:10.3390/molecules25061385)
Supplement: Supplementary file 1 [file molecules-25-01385-s001.pdf]

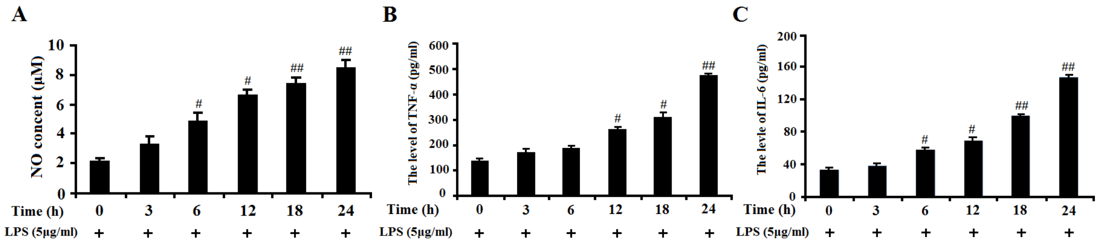

Figure 1. Effects of LPS on inflammatory responses in time-dependence in A549 cells. A549 cells were pretreated with LPS (5μg/mL) for 24h. A: The level of NO was measured in the culture medium by Griess reagents. B-C: The levels of TNF-α, IL-6 were detected in the culture medium by ELISA kits.
